# Supplementary figures and images for: Desikan-Killiany-Tourville Atlas Compatible Version of M-CRIB Neonatal Parcellated Whole Brain Atlas: The M-CRIB 2.0
Source: Front Neurosci. 2019 Feb 5;13:34. doi: 10.3389/fnins.2019.00034 (PMC6371012; doi:10.3389/fnins.2019.00034)

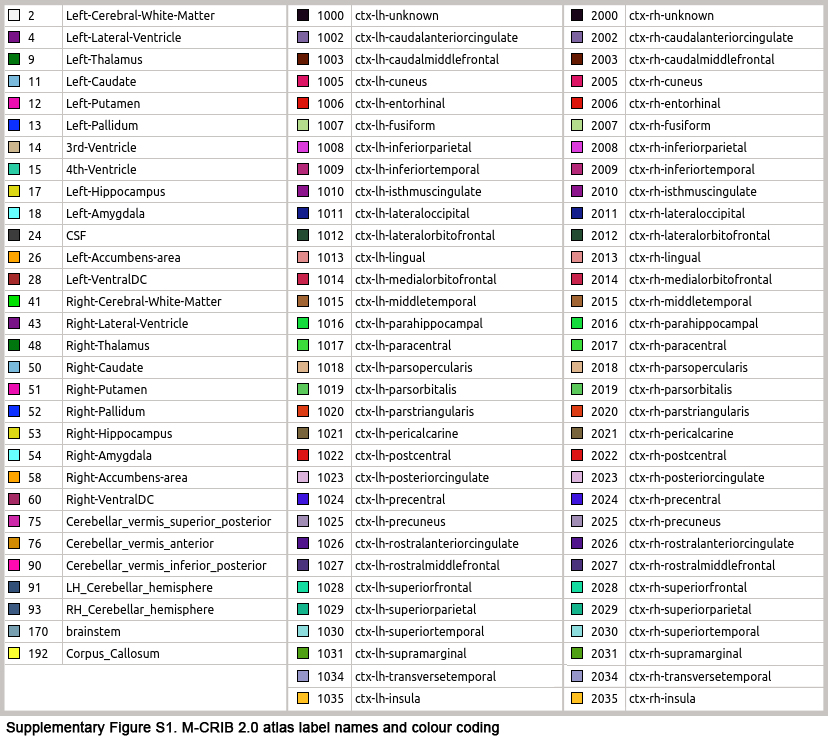

Supplement: Supplementary file 1 [file Image_1.JPEG]
